# Results of the Cologne Corona Surveillance (CoCoS) project– a cross-sectional study: survey data on risk factors of SARS-CoV-2 infection, and moderate-to-severe course in primarily immunized adults

**DOI:** 10.1186/s12889-024-17958-4

**Published:** 2024-02-21

**Authors:** Max Oberste, Teodora Asenova, Angela Ernst, Kija Shah-Hosseini, Nadja Schnörch, Michael Buess, Kerstin Daniela Rosenberger, Annelene Kossow, Felix Dewald, Florian Neuhann, Martin Hellmich

**Affiliations:** 1 Institute of Medical Statistics and Computational Biology, Medical Faculty and University Hospital of Cologne, University of Cologne, Robert-Koch-Straße 10, 50931 Cologne, Germany; 2 Cologne Health Authority, Cologne, Germany; 3 Institute of Hygiene, University Hospital of Muenster, University Muenster, Robert-Koch-Straße 49, 48149 Muenster, Germany; 4 Heidelberg Institute of Global Health, University Heidelberg, Heidelberg, Germany; 5 School of Medicine and Clinical Sciences, Levy Mwanawasa Medical University, Lusaka, Zambia; 6 Institute of Virology, Medical Faculty and University Hospital of Cologne, University of Cologne, Fürst-Pückler-Straße 56, 50935 Cologne, Germany

**Keywords:** Moderate-to-severe breakthrough infection, Booster vaccination, Age, Female sex, Smoking, Chronic lung disease, Infection severity

## Abstract

**Background:**

Amidst the COVID-19 pandemic, vaccination has been a crucial strategy for mitigating transmission and disease severity. However, vaccine-effectiveness may be influenced by various factors, including booster vaccination, as well as personal factors such as age, sex, BMI, smoking, and comorbidities. To investigate the potential effects of these factors on SARS-CoV-2 infection and disease severity, we analyzed data from the third round of the Cologne Corona Surveillance (CoCoS) project, a large cross-sectional survey.

**Methods:**

The study was conducted mid-February to mid-March 2022 in Cologne, Germany. A random sample of 10,000 residents aged 18 years and older were invited to participate in an online survey. Information on participants’ demographics (age, sex), SARS-CoV-2 infections, vaccination status, smoking, and preexisting medical conditions were collected. The outcomes of the study were: (1) the occurrence of SARS-CoV-2 infection despite vaccination (breakthrough infection) and (2) the occurrence of moderate-to-severe disease as a result of a breakthrough infection. Cox proportional-hazards regression was used to investigate possible associations between the presence/absence of booster vaccination, personal factors and the occurrence of SARS-CoV-2 infection. Associations with moderate-to-severe infection were analyzed using the Fine and Gray subdistribution hazard model.

**Results:**

A sample of 2,991 residents responded to the questionnaire. A total of 2,623 primary immunized participants were included in the analysis of breakthrough infection and 2,618 in the analysis of SARS-CoV-2 infection severity after exclusions due to incomplete data. The multivariable results show that booster vaccination (HR = 0.613, 95%CI 0.415–0.823) and older age (HR = 0.974, 95%CI 0.966–0.981) were associated with a reduced hazard of breakthrough infection. Regarding the severity of breakthrough infection, older age was associated with a lower risk of moderate-to-severe breakthrough infection (HR = 0.962, 95%CI0.949–0.977). Female sex (HR = 2.570, 95%CI1.435–4.603), smoking (HR = 1.965, 95%CI1.147–3.367) and the presence of chronic lung disease (HR = 2.826, 95%CI1.465–5.450) were associated with an increased hazard of moderate-to-severe breakthrough infection.

**Conclusion:**

The results provide a first indication of which factors may be associated with SARS-CoV-2 breakthrough infection and moderate-to-severe course of infection despite vaccination. However, the retrospective nature of the study and risk of bias in the reporting of breakthrough infection severity limit the strength of the results.

**Trial registration:**

DRKS.de, *German Clinical Trials Register* (DRKS), Identifier: DRKS00024046, Registered on 25 February 2021.

**Supplementary Information:**

The online version contains supplementary material available at 10.1186/s12889-024-17958-4.

## Introduction

On March 11, 2020, few months after its outbreak in Wuhan (China), the World Health Organization (WHO) declared COVID-19 a pandemic. By the time of writing in March 2023 over 759 million confirmed cases and over 6.8 million deaths worldwide have been caused by the ‘Severe acute respiratory syndrome coronavirus type 2’ (SARS-CoV-2) [1]. Germany counted more than 38 million cases and 168 thousand COVID-19-associated deaths by March 2023 [1].

A major contribution to preventing infections was made by the SARS-CoV-2-vaccines [2]. Previous studies have shown that vaccinated individuals have a lower viral load, experience fewer symptoms, and are at a significantly lower risk of transmitting the disease [3]. At the time the here presented study was conducted in mid-February to mid-March 2022, five vaccines namely BNT162b2 (mRNA vaccine by Pfizer-BioNTech), mRNA-1273 (mRNA vaccine by Moderna), Ad26.COV2.S (adenoviral vector vaccine by Janssen), NVX-CoV2373 (protein-based vaccine by Novavax) and ChAdOx1 (adenoviral vector vaccine by AstraZeneca) were available and approved for use in Germany [4]. By then, more than 171 million vaccine doses had been administered to over 63 million people in Germany, with more than 85,5% of the adult population (age 18 or older) having reached full vaccination status as defined by the Paul Ehrlich Institute in January 2022 (Primary immunization with two single doses of the above vaccines) [5, 6].

The vaccines licensed for use in Germany have been shown to be highly protective against SARS-CoV-2 infection [7, 8]. However, infections with SARS-CoV-2 do occur despite vaccination, and these cases are referred to as breakthrough infections [9, 10]. Even more important than protection against infection appears to be protection against a severe course of SARS-CoV-2 infection. Studies suggest a protective effect of vaccination against severe disease in the case of breakthrough infection [11]. However, severe disease courses occur despite vaccination [12, 13]. Regular booster vaccinations are intended to maintain a high level of protection and counteract a decline in vaccine efficacy [14]. Current recommendations are to revaccinate against SARS-CoV-2 infection after 3–6 months to maintain a high level of protection [15]. The effectiveness of booster vaccination is the subject of intensive scientific research [16–18]. 

In addition to booster vaccination, other factors are under discussion to influence the risk for vaccine breakthrough and severe COVID-19 disease progression despite vaccination. These include older age, smoking, and preexisting conditions such as obesity, chronic lung disease, cardiovascular disease, immunodeficiency, or cancer [12, 19]. It is important to identify the factors that increase the risk of vaccine breakthrough and severe COVID-19 disease despite vaccination, as this knowledge will allow more targeted protection beyond vaccination and further reduce the risk of SARS-CoV-2 infection, which is fatal in the worst case.

The primary objectives of this study were (1) to demonstrate a possible association between booster vaccination and the occurrence of breakthrough infection and (2) to investigate a possible association between booster vaccination and the severity of breakthrough infection. Secondary objectives of this study were to investigate possible associations of age, smoking, and pre-existing medical conditions with the occurrence of SARS-CoV-2 breakthrough infection and the severity of breakthrough infection. For this purpose, we used data from the third round of the Cologne Corona Surveillance (CoCoS) project, a large cross-sectional survey.

## Methods

### Setting

The CoCoS project was conducted in Cologne, a west German city situated in the federal state of North Rhine-Westphalia. With a population approaching 1.1 million, Cologne is the fourth-largest city in Germany. The average age of Cologne’s inhabitants approximates 42 years, however around 17.5% of the citizens are older than 65 years, considered as a vulnerable age group for a severe COVID-19 [20]. At the time of implementation of the third round of the CoCoS project, there were over 268,000 officially reported cases in Cologne and 1012 people had already died from or with SARS-CoV-2 in Cologne [21]. At that time, 85.4% of the adult population of Cologne had achieved primary immunization against COVID-19 [5, 6].

### Study design

The study reported here is cross-sectional. It represents a further development of earlier surveys conducted as part of CoCoS project [22–24]. The procedure was approved in advance by the Ethics Committee of the Medical Faculty of the University of Cologne and the Ethics Committee of the North Rhine Medical Association. The study was also registered in the German Clinical Trials Register (identifier: DRKS00024046). The survey was conducted from February 15 to March 15, 2022, a duration of exactly 4 weeks.

### Sample and setting

To participate in the survey, the following inclusion criteria applied: residence in Cologne and an age of 18 years or older. Exclusion criteria were: not resident in Cologne, under 18 years of age, or unable to give consent. We restricted the analysis of the associations between absence or presence of booster vaccination/age/smoking/preexisting medical conditions and SARS-CoV-2 infection/moderate-to-severe disease progression to those participants who had already completed their primary immunization and reported no SARS-CoV-2 infection prior to completing their primary immunization.

### Study procedures and data collection

A random sample of 10,000 Cologne residents was drawn from the municipal-registration office using a random generator in the official registration management program (MESO, HSH Soft- und Hardware Vertriebs GmbH, 16,356 Ahrensfelde OT Lindenberg). Participants were invited to partake in the study by mail on February 15, 2022. The invitation letter contained basic information about the purpose of the study and a printed QR code. Using this code, or alternatively the link that was also printed, participants were able to access the study homepage using their PC, tablet or smartphone. The homepage was kept as simple as possible in order to attract even citizens with little Internet experience to participate. First, the preferred language was queried. The choices were German, English and Turkish. Before starting the online questionnaire, participants were provided with comprehensive information about the study, e.g. about data protection, the approximate time needed to take part and the right to withdraw from participation at any time and to withdraw the data provided. Participants could then give their consent to participate by clicking on a button. The questionnaire was available for completion from February 15 to March 15, 2022. On February 24, 2022, all participants, who had not submitted a response yet received a reminder letter.

### Questionnaire

The online questionnaire first collected information on age, sex, height and weight. Subsequently, the participants were asked about medical conditions that had already existed prior to a possible SARS-CoV-2 infection. They were asked about their current smoking habits, the presence of chronic lung disease, cardiovascular disease, immunodeficiency and/or cancer. Participants were then asked about SARS-CoV-2 infections they had experienced and whether they had been vaccinated against COVID-19. If participants indicated that they had already had one or more SARS-CoV-2 infections, they were asked when these had occurred and what symptoms they had experienced. Regarding COVID-19 vaccination, participants were asked to indicate the date of the first vaccination, the date of the second vaccination, and, if applicable, the date of the booster vaccination.

### Definitions

SARS-CoV-2 infection was considered as such only when confirmed by a positive RT-qPCR test. SARS-CoV-2 infection despite primary immunization was considered a breakthrough infection. Infection severity among participants who reported a prior SARS-CoV-2 infection was assessed using the clinical spectrum classification criteria established by the U.S. National Institute of Health. According to this classification, SARS-CoV-2 infection is no longer considered mild when symptoms include shortness of breath or dyspnea. Further differentiation of disease progression into moderate, severe or critical is based on clinical parameters such as pulse oximetry oxygen saturation, respiratory rate and the presence of respiratory failure [25]. If participants reported that symptoms ranging from shortness of breath to dyspnea were present during their SARS-CoV-2 infection, the course was classified as moderate-to-severe. If participants reported the absence of this symptom, the course was classified as mild.

Primary immunization was considered to have been achieved if respondents reported having received two doses of a COVID-19 vaccine (Comirnaty (BioNTech/Pfizer), Spikevax (Moderna), Vaxzevria (AstraZeneca), Janssen (Johnson & Johnson), Nuvaxovid (Novavax) or a combination thereof, with the last dose having been administered at least 14 days before SARS-CoV-2 infection onset. The presence of booster vaccination was defined as a third dose of a COVID-19 vaccine [15, 26] given at least 7 days prior to infection. The interval of at least 14 days between primary immunization and infection, or 7 days between booster vaccination and infection, was chosen because studies show that the full effect of primary immunization does not occur until at least 14 days after the second dose [26], and the full effect of booster vaccination does not occur until 7 days after the booster [16, 26].

Smoking referred to participants’ smoking habits by the time of the questionnaire. Chronic lung disease included asthma, chronic bronchitis, chronic obstructive pulmonary disease (COPD) or emphysema. Cardiovascular disease identified chronic cardiovascular disorders, such as hypertension, coronary heart disease, heart insufficiency, irregular heartbeat, previous heartattack or stroke. A status of immunodeficiency was assumed if a primary immune disorder, transplantation of organ, chemotherapy or immunosuppressive medication such as cortisone was stated. Participants were required to report a cancer diagnosis of any type if they were currently undergoing treatment or had received treatment within the past year.

### Statistical analysis

We used the Cox proportional-hazards model to calculate the hazard ratios and 95% confidence intervals (CIs) of association between the absence or presence of SARS-CoV-2 booster vaccination and breakthrough infection. The variable “booster vaccination” was included in the model as a time-dependent covariate. As a time-scale defining time to infection, we used the date of second vaccination to the date of booster vaccination, infection, or participation in the survey, whichever occurred first. Hereafter, this time-scale will be referred to as “calendar time”. In addition to the booster variable, participants’ age at study entry, sex, Body-Mass-Index (BMI), smoking, presence of chronic lung disease, cardiovascular disease, immunodeficiency, and/or cancer were included in the model. Analyses were first univariable and then multivariable. In the multivariable analysis, backward selection (based on the Akaike Information Criterion (AIC)) from a pool of candidate variables was used to build a parsimonious model. To analyze associations between the aforementioned variables and severity of progression, an analysis of competing risks was performed using the Fine and Gray subdistribution hazard model. A moderate-to-severe course of SARS-CoV-2 infection is in a competing risk situation to a mild course of SARS-CoV-2 infection. Here, too, the booster variable was included in the model as a time-dependent covariate, and calendar time was again used as the time-scale. Variable selection here was analogous to the procedure described above.

Only those variables that had at least five events, were included in the analysis. Multicollinearity was assessed by examining the variance inflation factors (VIF) of all included variables. Any variable with a VIF greater than five was to be excluded.

Statistical analysis was performed using SPSS Statistics (IBM Corp., Version 28.0, Armonk, NY, USA) and the statistical software R (R Foundation for Statistical Computing, Version 4.2.1, Vienna, Austria).

## Results

### Sample characteristics

**Fig. 1:**
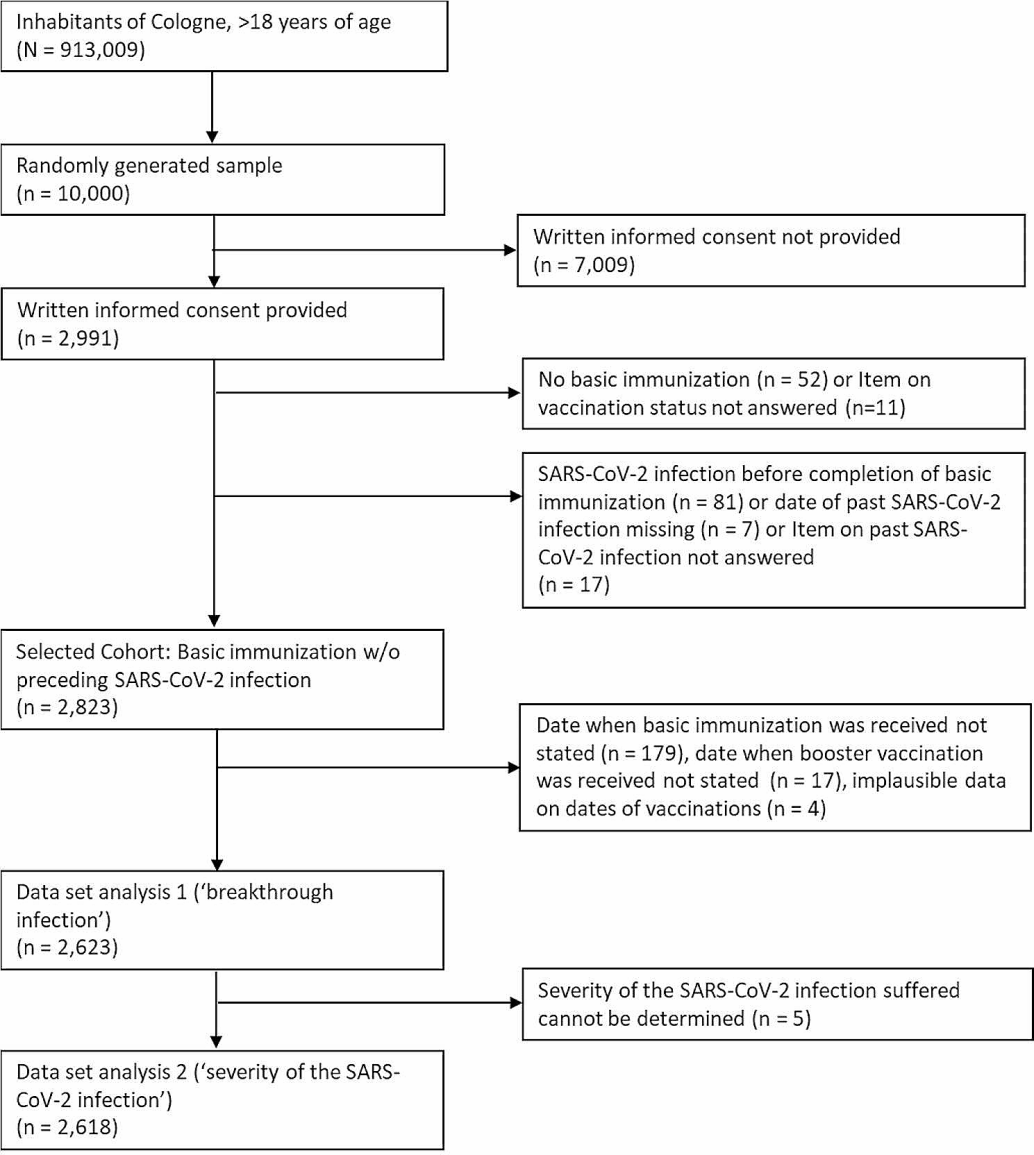
Overview of the recruitment for the third round of the CoCoS project, the selection of the cohorts up to the datasets eventually analyzed here

The flow chart in Fig. 1 provides an overview of the recruitment for the third round of the CoCoS project, the selection of the cohorts up to the datasets eventually analyzed here. Of the 10,000 Cologne residents who were contacted, 2,991 (29.9%) gave their consent to participate in the survey. Of these, 2,823 individuals stated that they had already received primary immunization against SARS-CoV-2 infection and had not contracted a SARS-CoV-2 infection prior to this primary immunization. However, of this selected cohort, an additional 200 individuals were excluded from the analyses due to missing or implausible time data on primary immunization or booster vaccination. The data set used for the analysis of associations with breakthrough infection thus included 2,623 primarily immunized adult residents of Cologne. With regard to the analysis of the severity of the SARS-CoV-2 breakthrough infection, five more participants had to be excluded from the analysis. They reported that they had suffered a SARS-CoV-2 infection after their primary immunization, but at the same time did not answer the questions necessary to classify the severity of the SARS-CoV-2 infection. Accordingly, the dataset used for the analysis of associations with the severity of SARS-CoV-2 breakthrough infection included 2,618 adult residents of Cologne who had received primary immunization. Participants’ sociodemographic characteristics, vaccination status, smoking habits, and preexisting medical conditions subdivided by outcome are summarized in Table 1.

### Analysis of associations between vaccination status, age, sex, body-mass-index, smoking habits, preexisting medical conditions and breakthrough infection

Of the 2,623 primarily immunized adult Cologne residents included in this analysis, 287 (10.94%) reported a breakthrough infection. The date of first reported breakthrough infection in the cohort analyzed here was 1 April 2021. Among the participants who received a booster dose in addition to the primary immunization, the mean time between the primary immunization and the booster dose was 166.7 days (SD = 34.3). The results of both univariable and multivariable cox proportional hazard regression analysis with calendar time as time-scale and dependent variable breakthrough infection are presented in Table 2 and Supplementary Fig. 1. In the multivariable analysis, 2,566 participants with complete cases for the variables used here could be included. Of 2,623 participants, 51 participants were excluded from the multivariable analysis, five because of missing information on smoking behavior, and one participant because of incomplete information on body mass index and one participant because of incomplete information on body mass index and missing information on smoking behavior. The multicollinearity test did not reveal any indication to take further steps. The results of the multicollinearity assessment are included in the Supplementary Material to this article, specifically in Supplementary Table 1.

### Association between vaccination status and breakthrough infection

**Fig. 2:**
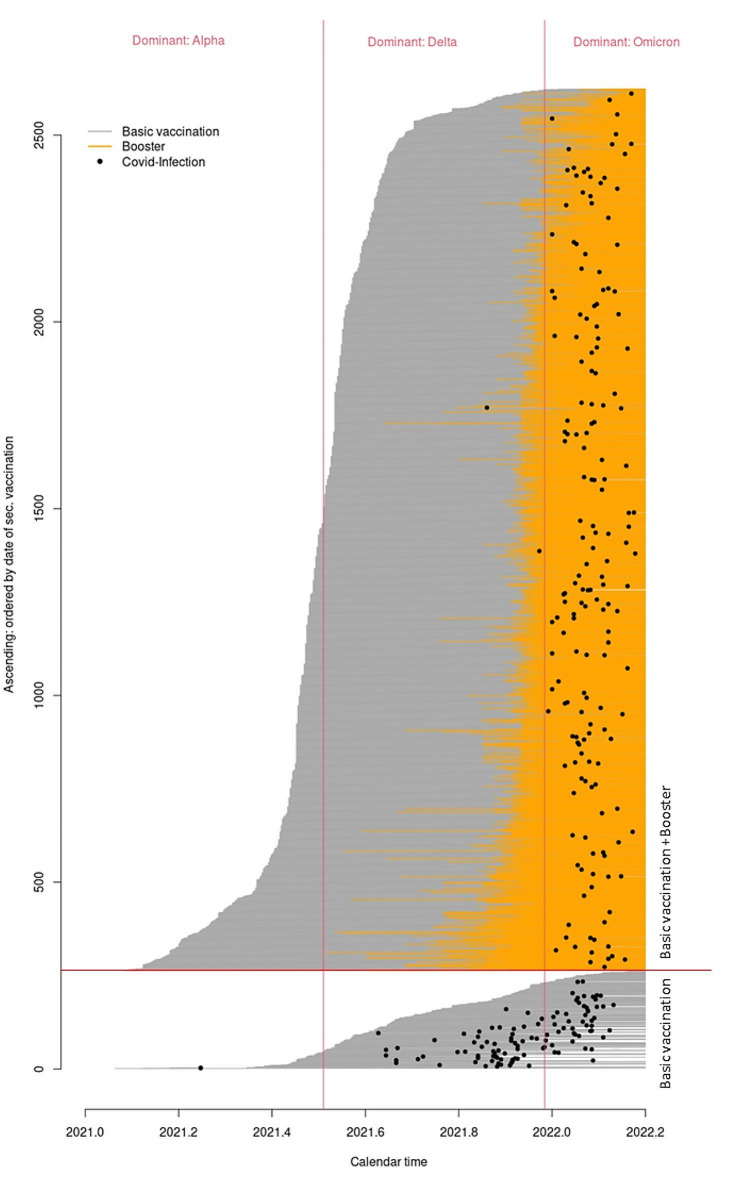
Lexis plot– the time course for each participant from the date of primary immunization to possible booster vaccination (change from gray to orange) and/or possible SARS-CoV-2 infection (dot) and/or, in the case of no infection, end of the survey on 15 March 2022

The vast majority of the cohort analyzed here had already received booster vaccination beyond primary immunization against SARS-CoV-2 infection at the time of the survey. Among participants censored (no infection) in the Cox proportional-hazards regression analysis, the proportion who had already received an initial booster vaccination against SARS-CoV-2 infection beyond the primary immunization was 93.6%. Of the 287 participants who reported a breakthrough infection, 61.0% reported that they had received a booster dose of the SARS-CoV-2 vaccine at some point in the past. The Lexis plot in Fig. 2 shows the time course for each participant from the date of primary immunization to possible booster vaccination and possible SARS-CoV-2 infection. The Lexis plot ends for participants either with a SARS-CoV-2 infection or, in the case of no infection, at the end of the survey on 15 March 2022. Figure 2 also shows that the time of the participants’ booster vaccination was during the cold season and the phase of changing predominant variants from Delta to Omicron [24]. In the univariable analysis with vaccination status as a time-dependent covariate and calendar time as the time axis, a statistically significant association between vaccination status and hazard of breakthrough infection was shown. Additional booster vaccination against SARS-CoV-2 infection was associated with a significantly reduced hazard of breakthrough infection compared with primary immunization alone (HR = 0.483, 95% CI 0.356–0.652, *p* <.001). This finding could be confirmed in the multivariable analysis (HR = 0.613, 95% CI 0.415–0.823, *p* =.002).

### Associations between age, sex, body-mass-index, smoking habits, preexisting medical conditions and breakthrough infection

While censored participants (without breakthrough infection at the time of the survey) were on average 50.44 years old (SD = 16.88), participants who reported a breakthrough infection were significantly younger, on average 42.52 years old (SD = 15.03). Univariable Cox regression showed a statistically significant association between age and breakthrough infection, with a decreasing risk with increasing age (HR = 0.972, 95% CI 0.965–0.979, *p* = < 0.001). The significant association between younger age and breakthrough infection also remained in the multivariable Cox proportional-hazards model. In the multivariable model, one additional year of life reduced the hazard of breakthrough infection by 2.6%, which was statistically significant (HR = 0.974, 95% CI 0.966–0.981, *p* = < 0.001).

Among participants who reported a breakthrough infection, the proportion of women was higher than among censored participants without a breakthrough infection (59.2% vs. 53.0%). However, there was no statistically significant association between participant sex and breakthrough infection in either univariable (HR = 1.260, 95% CI 0.995–1.549, *p* =.055) or multivariable (HR = 1.231, 95% CI 0.970–1.561, *p* =.087) analysis. With regard to BMI, participants with a breakthrough infection were not different from censored participants who did not report a breakthrough infection (25.34 ± 4.96 versus 25.46 ± 4.71). In line with this, no statistically significant association was found between participants’ BMI and breakthrough infection in either univariable (HR = 0.995, 95% CI 0.971–1.020, *p* =.699) or multivariable (HR = 1.015, 95% CI 0.991–1.040, *p* =.214) analysis. The percentage of smokers was 2.6% higher among participants who reported a breakthrough infection than among those who did not (23.0% vs. 20.4%). Cox regression showed no statistically significant association between smoking behavior and hazard of breakthrough infection, either univariable (HR = 1.154, 95% CI 0.876–1.519, *p* =.309) or multivariable (variable was excluded from the model during stepwise backward selection).

In terms of pre-existing medical problems, the group of participants with breakthrough infection had a lower percentage of participants with chronic lung disease (6.3% vs. 8.6%), cardiovascular disease (17.1% vs. 24.7%) or cancer (1.7% vs. 2.5%) than the group of participants without breakthrough infection. The two groups were similar with regard to immunodeficiency (2.8% vs. 2.6%). Cox regression (univariable) showed a statistically significant association with breakthrough infection only for pre-existing cardiovascular disease. Univariable analysis showed that the presence of a cardiovascular disease was associated with a significantly lower risk of breakthrough infection (HR = 0.636, 95% CI 0.467–0.864, *p* =.004). In the multivariable analysis, all variables concerning preexisting medical conditions were excluded from the model during stepwise backward selection.

**Table 1:** Participants’ sociodemographic characteristics, vaccination status, smoking habits, and pre-existing medical conditions subdivided by outcome

	Dataset 1 (*n* = 2,623)		SARS-CoV-2 infection (*n* = 287)		Dataset 2 (*n* = 2,618)		Moderate-to-severe SARS-CoV-2 infection (*n* = 60)		Mild SARS-CoV-2 infection (*n* = 222)		Censored (*n* = 2,336)	
Age* (mean, SD)	49.57	16.89	42.52	15.03	49.59	16.86	40.33	13.13	43.18	15.44	50.44	16.88
**Age groups**												
18–34 years (n, %)	619	23.6%	95	33.1%	617	23.6%	22	36.7%	71	32.0%	524	22.4%
35–59 years old (n, %)	1,226	46.7%	152	53.0%	1,223	46.7%	34	56.7%	115	51.8%	1,074	46.0%
60–74 years old (n, %)	576	22.0%	34	11.8%	576	22.0%	4	6.7%	30	13.5%	542	23.2%
75 years or older (n, %)	202	7.7%	6	2.1%	202	7.7%	0	0.0%	6	2.7%	196	8.4%
Missing values (n, %)	0	0.0%	0	0.0%	0	0.0%	0	0.0%	0	0.0%	0	0.0%
Sex*												
Female (n, %)	1,408	53.7%	170	59.2%	1,404	53.6%	45	75.0%	121	54.5%	1,238	53.0%
Male (n, %)	1,215	46.3%	117	40.8%	1,214	46.4%	15	25.0%	101	45.5%	1,098	47.0%
Missing values (n, %)	0	0.0%	0	0.0%	0	0.0%	0	0.0%	0	0.0%	0.0	0.0%
**BodyMassIndex** (mean, SD)	25.45	4.74	25.34	4.96	25.54	4.74	25.74	5.99	25.27	4.70	25.46	4.71
**BodyMassIndex groups**												
< 18.5 (n, %)	41	1.6%	9	3.1%	41	1.6%	3	5.0%	6	2.7%	32	1.4%
18.5–24.9 (n, %)	1,351	51.5%	145	50.5%	1,348	51.5%	29	48.3%	113	50.9%	1,206	51.6%
25-29.9 (n, %)	799	30.5%	85	29.6%	798	30.5%	15	25.0%	69	31.1%	714	30.6%
≥ 30 (n, %)	380	14.5%	46	16.0%	380	14.5%	13	21.7%	33	14.9%	334	14.3%
Missing values (n, %)	52	2.0%	2	0.7%	51	1.9%	0	0.0%	1	0.5%	50	2.1%
**Vaccination status** ^**#**^												
Primary immunization w/o booster (n, %)	262	10.0%	112	39.0%	258	9.9%	21	35.0%	87	39.2%	150	6.4%
Primary immunization w/ booster (n, %)	2,361	90.0%	175	61.0%	2,360	90.1%	39	65.0%	135	60.8%	2,186	93.6%
Missing values	0	0.0%	0	0.0%	0	0.0%	0	0.0%	0	0.0%	0	0.0%
**Smoking**												
Yes (n, %)	543	20.7%	66	23.0%	541	20.7%	20	33.3%	44	19.8%	477	20.4%
No (n, %)	2,074	79.1%	221	77.0%	2,071	79.1%	40	66.7%	178	80.2%	1,853	79.3%
Missing values (n, %)	6	0.2%	0	0.0%	6	0.2%	0	0.0%	0	0.0%	6	0.3%
**Chronic lung disease**												
Yes (n, %)	218	8.3%	18	6.3%	216	8.3%	11	18.3%	5	2.3%	200	8.6%
No (n, %)	2,405	91.7%	269	93.7%	2,402	91.7%	49	81.7%	217	97.7%	2,136	91.4%
Missing values (n, %)	0	0.0%	0	0.0%	0	0.0%	0	0.0%	0	0.0%	0	0.0%
**Cardiovascular disease**												
Yes (n, %)	627	23.9%	49	17.1%	626	23.9%	11	18.3%	37	16.7%	578	24.7%
No (n, %)	1,996	76.1%	238	82.9%	1,992	76.1%	49	81.7%	185	83.3%	1,758	75.3%
Missing values (n, %)	0	0.0%	0	0.0%	0	0.0%	0	0.0%	0	0.0%	0	0.0%
**Immunodeficiency**												
Yes (n, %)	69	2.6%	8	2.8%	68	2.6%	0	0.0%	7	3.2%	61	2.6%
No (n, %)	2,554	97.4%	279	97.2%	2,550	97.4%	60	100.0%	215	96.8%	2,275	97.4%
Missing values^**^ (n, %)	0	0.0%	0	0.0%	0	0.0%	0	0.0%	0	0.0%	0	0.0%
**Cancer present or treated in the last year**												
Yes (n, %)	64	2.4%	5	1.7%	64	2.4%	1	1,7%	0	0.0%	59	2.5%
No (n, %)	2,559	97.6%	282	98.3%	2,554	97.6%	59	98.3%	222	100.0%	2,277	97.5%
Missing values (n, %)	0	0.0%	0	0.0%	0	0.0%	0	0.0%	0	0.0%	0	0.0%

SD– standard deviation, n - frequency, % - percentage

* Information obtained directly from the population register

^#^ time-dependent analysis

**Table 2:** Associations with vaccination breakthroughs– results of univariable and multivariable Cox proportional hazards survival regression

Variables	Univariable analysis				Multivariable analysis (*N* = 2,566)		
	*N*	Hazard Ratio	95% CI	*p*	Adjusted Hazard Ratio	95% CI	*p*
**Age** ^**1**^	2,623	0.972	0.965–0.979	< 0.001	0.974	0.966–0.981	< 0.001
**Sex**							
Male	1,215	Reference			Reference		
Female	1,408	1.260	0.995–1.549	0.055	1.231	0.970–1.561	0.087
**Body Mass Index**	2,571	0.995	0.971–1.020	0.699	1.015	0.991–1.040	0.214
Vaccination status*							
Primary immunization	262	Reference			Reference		
Primary immunization + booster	2,361	0.483	0.356–0.652	< 0.001	0.613	0.451–0.832	0.002
**Smoking**							
No smoker	2,074	Reference					
Smoker	543	1.154	0.876–1.519	0.309			
**Chronic lung disease**							
No	2,405	Reference					
Yes	218	0.730	0.453–1.177	0.197			
**Cardiovascular disease**							
No	1,996	Reference					
Yes	627	0.636	0.467–0.864	0.004			
**Immunodeficiency**							
No	2,554	Reference					
Yes	69	1.030	0.510–2.080	0.934			
**Cancer**							
No	2,559	Reference					
Yes	64	0.683	0.282–1.654	0.399			

* The booster variable was included in the model as a time-dependent covariate

### Analysis of associations between vaccination status, age, sex, body-mass-index, smoking habits, pre-existing medical conditions and severity of SARS-CoV-2 breakthrough infection

Of the 2,618 adult Cologne residents with primary immunization included in this analysis, 60 reported to have suffered from a moderate-to-severe breakthrough infection and 222 reported to have suffered from a mild breakthrough infection. The results of the univariable and multivariable analyses of competing risks are summarized in Table 3 and Supplementary Fig. 2. The multivariable analysis included data from 2,562 individuals. Of 2,618 participants, 50 participants were excluded from the multivariable analysis, five because of missing information on smoking behavior, and one participant because of incomplete information on body mass index and one participant because of incomplete information on body mass index and missing information on smoking behavior. The test for multicollinearity did not reveal any need for corrections. The results of this multicollinearity analysis are presented in Supplementary Table 2 attached to this article.

### Associations between vaccination status and severity of SARS-CoV-2 breakthrough infection

The percentage of censored participants who had received an additional booster vaccination against SARS-CoV-2 at the time of the survey was 93.6%. Among participants who reported moderate-to-severe breakthrough infection, the percentage who had received a booster dose was 65.0%. Among those who reported a mild breakthrough infection, the percentage who had received a booster dose was 60.8%. In univariable analysis of competing risks with vaccination status as a time-dependent covariate and calendar time as a time axis, additional booster vaccination against SARS-CoV-2 infection was associated with a lower hazard of moderate-to-severe breakthrough infection than primary immunization alone. However, this association did not reach statistical significance (HR = 0.819, 95% CI 0.457–1.468, *p* =.502). In the multivariable analysis, the variable vaccination status was excluded from the model during stepwise backward selection.

### Associations between age, sex, BMI, smoking habits, preexisting medical conditions and severity of SARS-CoV-2 breakthrough infection

Participants who reported moderate-to-severe breakthrough infection (40.33 ± 13.13 years) and participants who reported mild breakthrough infection (43.18 ± 15.44 years) were on average significantly younger than censored participants without breakthrough infection (50.44 ± 16.88 years). Univariable analysis of competing risks showed a statistically significant association between younger age and moderate-to-severe breakthrough infection (HR = 0.964, 95% CI 0.950–0.978, *p* = < 0.001). This significant association remained stable in multivariable analysis. In the multivariable Fine and Gray subdistribution hazard regression, one additional year of life reduced the hazard of a moderate-to-severe breakthrough infection by 3.8%, which was statistically significant (HR = 0.962, 95% CI 0.949–0.977, *p* = < 0.001).

Among participants reporting moderate-to-severe SARS-CoV-2 breakthrough infection (75.0%), the proportion of women was significantly higher than among participants reporting mild breakthrough infection (54.5%) or censored participants with no breakthrough infection (53.0%). There was a statistically significant association between participant sex and the hazard of moderate-to-severe SARS-CoV-2 breakthrough infection. With a Hazard Ratio of 2.524 (95% CI 1.404–4.537) in univariable analysis and a Hazard Ratio of 2.570 (95% CI 1.435–4.603) in multivariable analysis, women had a significantly increased risk of moderate-to-severe breakthrough infection compared with men. The mean BMI of participants reporting moderate-to-severe (25.74 kg/m^2^), mild breakthrough infection (25.27 kg/m^2^), or censored participants (25.46 kg/m^2^) differed insignificantly from each other. In line with this finding, BMI neither showed significant association with the severity of SARS-CoV-2 breakthrough infection in the univariable (HR = 1.014, 95% CI 0.952–1.079, *p* =.669) nor in the multivariable (HR = 1.032, 95% CI 0.982–1.084, *p* =.211) Fine and Gray subdistribution hazard regression. The percentage of smokers among participants who reported moderate-to-severe SARS-CoV-2 breakthrough infection (33.3%) was strikingly higher than among participants who reported mild breakthrough infection (19.8%) or among censored participants without breakthrough infection (20.4%). In the univariable competing hazards analysis, a significant association was found between smoking behavior and the risk of SARS-CoV-2 breakthrough infection. Smokers had a significantly increased hazard of moderate-to-severe breakthrough infection compared with non-smokers (HR = 1.978, 95% CI 1.156–3.384, *p* =.013). This finding was confirmed in multivariable analysis (HR = 1.965, 95% CI 1.147–3.367, *p* =.012). With regard to pre-existing medical conditions, the variables immunodeficiency and cancer were not included in the analysis because of the 60 participants who reported moderate-to-severe breakthrough infection, none or only one had such pre-existing medical conditions. Among participants reporting moderate-to-severe breakthrough infection, the prevalence of chronic lung disease was significantly higher (18.3%) than among participants reporting mild breakthrough infection (2.3%) or among censored participants with no breakthrough infection (8.6%). Univariable (HR = 2.585, 95% CI 1.346–4.964, *p* =.004) and multivariable analysis (HR = 2.826, 95% CI 1.465–5.450, *p* =.002) confirmed an association between the presence of chronic lung disease and an increased risk of moderate-to-severe breakthrough infection. Pre-existing cardiovascular disease was proportionally less frequent in the moderate-to-severe group and in the mild breakthrough infection group than in the censored participants. However, univariable (HR = 0.723, 95% CI 0.376–1.392, *p* =.332) and multivariable analysis (the variable was excluded from the model using stepwise backward selection) showed no systematic association.

**Table 3:** Associations with moderate-to-severe course of COVID-19– results of univariable and multivariable Fine and Gray subdistribution hazard regression

Variables	Univariable analysis				Multivariable analysis (*N* = 2,562)		
	*N*	Hazard Ratio	95% CI	*p*	Adjusted Hazard Ratio	95% CI	*p*
**Age** ^**1**^	2,618	0.964	0.950–0.978	< 0.001	0.962	0.949–0.977	< 0.001
**Sex**							
Male	1,214	Reference			Reference		
Female	1,404	2.524	1.404–4.537	0.002	2.570	1.435–4.603	0.002
**Body Mass Index**	2,583	1.014	0.952–1.079	0.669	1.032	0.982–1.084	0.211
Vaccination status*							
Primary immunization	262	Reference					
Primary immunization + booster	2,361	0.819	0.457–1.468	0.502			
**Smoking**							
No smoker	2,071	Reference			Reference		
Smoker	541	1.978	1.156–3.384	0.013	1.965	1.147–3.367	0.012
**Chronic lung disease**							
No	2,405	Reference			Reference		
Yes	218	2.585	1.346–4.964	0.004	2.826	1.465–5.450	0.002
**Cardiovascular disease**							
No	1,996	Reference					
Yes	627	0.723	0.376–1.392	0.332			

* The booster variable was included in the model as a time-dependent covariate

## Discussion

The results presented above are based on data from more than 2,600 adults in Cologne who had completed the primary immunization schedule. In the cohort studied, breakthrough infections occurred with a relative frequency of 10.94% in the period from the start of the pandemic in January 2020 (the first known case of SARS-CoV-2 infection in Germany is dated 28 January 2020 [27]) to the study cut-off date of 15 March 2022. Our results suggest that younger age, lack of booster vaccination and the absence of cardiovascular disease are associated with vaccine breakthrough. Our results also suggest that younger age, female sex, smoking and chronic lung disease are associated with COVID-19 breakthrough infection of moderate-to-severe disease course.

The finding that additional booster vaccination is associated with a statistically significant lower hazard of breakthrough infection compared with primary immunization alone is consistent with the results of recent studies [28]. Booster vaccines have been shown to improve the immune response, inducing higher antibody levels and higher virus neutralization [29]. However, our results did not show a statistically significant advantage of booster vaccination over primary immunization alone in terms of severity of breakthrough infection. This finding differs from the results of other studies showing a high efficacy of booster vaccination in preventing severe COVID-19 infections [16]. As shown in Fig. 2, most booster vaccinations in our cohort were given during the cold season, when more and more severe infection can be expected [30, 31]. The change in the predominant SARS-CoV-2 variant in Germany from Delta- to Omicron in January 2022 [26] also coincides with most booster vaccinations in our cohort. These and other time-varying covariates should be controlled for in the present study by using calendar time as the time scale of the time-to-event regression models used. The use of calendar time as the time scale in the analysis performed here allows comparison between participants who received an additional booster vaccination and those who received only a primary immunization to be made on participants at risk at the same calendar time. Thus, the comparison is made under the same time-dependent conditions (e.g., SARS-CoV-2 incidence, predominant virus variant, time of the year), and time-varying covariates are unlikely to explain the lack of effect of booster vaccination on the risk of severe breakthrough infection. Lund and colleagues (2023) show in their article that the use of calendar time is the most effective way to control for bias due to time-varying covariates in studies of COVID-19 vaccine efficacy [32]. However, covariates such as virus variant can still confound results. This may be the case, for example, if vaccines lose significant efficacy against new virus variants and at the same time the number of new infections increases significantly.

Empirically, the additional booster vaccination showed an advantage over the primary immunization alone. The lack of statistical significance could be due to the sample size with a relatively small effect. The small effect of the booster vaccination compared to primary immunization could potentially be explained by the timing of the booster administration. The recommendation for booster vaccination is 3–6 months after primary immunization, as indicated in current guidelines [15], which is consistent with the majority of participants in our study receiving a booster within this time interval. However, it is important to note that the study was conducted amidst the emergence of the Omicron variant. As the majority of participants received a booster before this time, it is likely that they were vaccinated with older vaccines that were not specifically adapted to the Omicron variant [33]. The age of our participants showed a statistically significant association with the hazard of SARS-CoV-2 breakthrough infection. Younger age was associated with a higher likelihood of vaccine breakthrough. Studies on the process of infection have attributed a similar risk of disease to younger and older people, because the process of infection does not differ significantly between the two groups [34]. However, retrospective cohort studies such as this one, but also prospective cohort studies, repeatedly show that younger age is associated with a higher risk of infection [35, 36]. This has recently been shown specifically for breakthrough infections in adults with primary immunization against SARS-CoV-2 [37]. The main explanation given for these findings is that older people are more cautious and more likely to comply with preventive measures than younger people. According to literature this finding is equivocal with supportive [38] and contradicting results [39]. Surprisingly, our study also showed a positive association between younger age and the hazard of a breakthrough infection becoming moderate-to-severe. Prospective cohort studies with clinical symptom recording by professionally trained staff have shown the opposite. Higher rates of severe cases were found in older age groups [40]. Infectious disease studies suggest that a greater propensity to inflammation, an aged immune system and changes in the cytokine profile are associated with this higher susceptibility to severe outcomes in old age [34]. One reason why our results differ from these studies may be the classification of moderate-to-severe disease used here. Our participants were asked whether they experienced symptoms ranging from breathlessness to shortness of breath during their SARS-CoV-2 infection. It is well known from research on asthma patients and patients with other chronic lung diseases that older patients may downplay symptoms of breathlessness and shortness of breath and may even perceive them as less severe than younger people [41, 42]. This is even more true for early or mild symptoms of breathlessness [43].

Participant sex did not show a statistically significant association with the hazard of breakthrough infection. However, sex did show a statistically significant association with the severity of a breakthrough infection. Surprisingly, our study showed a higher risk of moderate-to-severe disease in women compared to men. This finding is at odds with the current state of research. Many epidemiological and infectious disease studies suggest that men are more likely to develop and die from severe COVID-19 [40, 44, 45]. As described above for age, the reason may be subjective reporting of severity as the presence of shortness of breath to dyspnea. Studies show that women report symptoms such as shortness of breath and dyspnea more frequently and more severely than men with the same lung function impairment [46, 47].

Our results found no significant association between participant’s BMI and the likelihood of a SARS-CoV-2 breakthrough infection or disease severity. This contradicts previous research that has identified a greater risk of severe COVID-19 in individuals with higher BMI and obesity, even after receiving basic vaccination [48, 49]. Excess body fat has been associated with a range of functional disorders, including local infiltration with immune cells, higher leptin levels and the release of pro-inflammatory cytokines that can negatively impact the function of other tissues in the body. These processes can exacerbate COVID-19 symptoms [50]. As mentioned earlier, we identified moderate-to-severe COVID-19 cases by the presence of dyspnea. However, the pro-inflammatory effects of adipose tissue are not restricted to the respiratory system but can also affect other organ systems involved in the progression of severity [50]. In order to gain a better understanding of how obesity affects COCID-19 severity, a comprehensive definition of severe infection that considers the impact of obesity on multiple organ systems and associated symptoms is necessary. Another possible explanation for the divergent findings could be the reliance on BMI as the sole measure for defining obesity. BMI has limitations, as it does not consider differences in body composition and distribution of body fat [51]. Therefore, higher BMI values alone may not provide an accurate representation of how obesity impacts COVID-19 severity. Other measures, such as waist circumference, waist-to-hip ratio or body fat percentage, may provide more precise measures of obesity and better predict the risk of severe COVID-19 outcomes [52].

Smoking behavior did not show a statistically significant association with the likelihood of vaccine breakthrough. However, smoking did show a statistically significant association with the likelihood of moderate-to-severe vaccine breakthrough. In our cohort, we found a significantly higher risk of moderate-to-severe breakthrough infection in smokers compared with non-smokers. This finding is consistent with other studies [53]. On a biological level, the greater susceptibility of smokers to severe outcomes is explained by increased systemic inflammation and increased viral replication in lung tissue as a result of smoking [54]. In addition, nicotine has been shown to increase the expression of angiotensin converting enzyme 2 (ACE-2) in human bronchial epithelial cells [55].

For three of the four pre-existing medical problems examined here, there was a univariable trend towards a lower risk of breakthrough infection. This may seem surprising at first sight. However, extra cautious behavior and strict adherence to hygiene measures may have compensated for a possible increased susceptibility. There is little research on whether people with pre-existing conditions have adapted their behavior in this way. However, early evidence suggests that this is the case. Regarding the severity of the breakthrough infection, there was a statistically significant association between the presence of chronic lung disease and the likelihood of a moderate-to-severe breakthrough infection. This is consistent with the results of other studies [56, 57] and is to be expected because SARS-CoV-2 initially attacks the respiratory system. This, together with chronic lung disease, can lead to acute respiratory distress syndrome [58]. It is important to inform people with chronic lung disease about the possible severity of infection despite vaccination, so that they can adjust their behavior to avoid infection.

The participation rate of the study (29.91%) is high, considering that participation was unrewarded (and of course voluntary). The questionnaire covered a wide range of areas and provided a detailed description of participant’s sociodemographic characteristics, potentially risk behaviors, vaccination status and health condition. Using calendar time as the time scale for statistical analysis allows comparison of participants in terms of reported infections who were at risk at the same calendar time. This controls for associations between variable expression and seasonal variation in infection numbers.

Our study is limited to the population aged 18 and older. Therefore, the results may not be generalizable to the entire population, including children and adolescents. Since most SARS-CoV-2 vaccines are now also applicable to and even recommended for the youth population [59], future studies could do a further investigation on associations with vaccine breakthrough and poor outcomes also including persons younger than 18 years. The questionnaire method of data collection entails a high risk of response bias. This is particularly true for the severity of breakthrough infection. When participants reported having an infection, they were asked whether they had experienced symptoms ranging from shortness of breath to dyspnea as part of that infection. However, as explained above, the perception and willingness to report this depends on a number of factors and is not an objective measure. In addition, the classification of severity based on the presence of shortness of breath to dyspnea only allows a very rough division of severity into moderate to severe. Although moderate infections are likely to be a distressing experience for those affected, they are by definition not life-threatening. Another notable limitation of our study is the digital divide bias. Despite efforts to facilitate access, participants who are comfortable using the internet may be systematically different from non-users. This may have resulted in older adults and those less familiar with the internet and digital media feeling less addressed. In addition, information provided by participants with limited digital experience may have introduced bias, due to typographical and other errors.

## Conclusion

The study provides insights into factors associated with breakthrough infections and their severity. It suggests that absence of booster vaccination and younger age are associated with vaccine breakthrough, while moderate-to-severe breakthrough infection is associated with smoking, chronic lung disease, younger age and female sex. These findings can help identify individuals who may be at higher risk of breakthrough infection and severe outcomes and inform targeted communication and vaccination strategies to mitigate the impact of COVID-19. However, due to risk of bias in the recording of severity of breakthrough infection, caution should be taken in interpreting and acting on these results.

### Electronic supplementary material

Below is the link to the electronic supplementary material.

Supplementary Material 1

## Data Availability

The datasets used and/or analysed within the current study are available from the corresponding author upon reasonable request.

## Abbreviations

SARS-CoV-2: Severe Acute Respiratory Syndrome-Coronavirus-2
COVID-19: Corona Virus Disease 19

## References

[1] WHO. WHO Coronavirus (COVID-19) Dashboard. 2023. https://covid19.who.int. Accessed 14 Mar 2023.

[2] National Institutes of Health. Prevention of SARS-CoV-2 infection. Coronavirus disease 2019 (COVID-19) treatment guidelines. 2022. https://www.covid19treatmentguidelines.nih.gov/overview/prevention-of-sars-cov-2/. Accessed 1 Mar 2023. 34003615

[3] Hsu L, Grüne B, Buess M, Joisten C, Klobucnik J, Nießen J (2021). COVID-19 breakthrough infections and transmission risk: real-World Data analyses from Germany’s Largest Public Health Department (Cologne). Vaccines.

[4] Robert Koch-Institut. Wöchentlicher Lagebericht des RKI zur Coronavirus-Krankheit-2019 (COVID-19). Berlin: Robert-Koch-Institut.; 2022. https://www.rki.de/DE/Content/InfAZ/N/Neuartiges_Coronavirus/Situationsberichte/Wochenbericht/Wochenbericht_2022-03-17.pdf?__blob=publicationFile Accessed 13 Feb 2023.

[5] Robert Koch-Institut. Fachgebiet 33. COVID-19-Impfungen in Deutschland (2022-03-16). Zenodo. 2022. 10.5281/zenodo.6361365. Accessed 1 Mar 2023.

[6] Paul-Ehrlich-Institut. Impfnachweis im Sinne der COVID-19-Schutzmaßnahmen-Ausnahmenverordnung. Paul-Ehrlich-Institut.; 2022. https://www.pei.de/SharedDocs/Downloads/DE/newsroom/dossiers/archiv-seite-impfnachweis/impfnachweis-covid-19-verordnungen-stand-10-03-2022.pdf?__blob=publicationFile&v=2 Accessed 1 Mar 2023.

[7] Abu-Raddad LJ, Chemaitelly H, Butt AA (2021). Effectiveness of the BNT162b2 Covid-19 vaccine against the B.1.1.7 and B.1.351 variants. N Engl J Med.

[8] Butt AA, Omer SB, Yan P, Shaikh OS, Mayr FB (2021). SARS-CoV-2 vaccine effectiveness in a high-risk National Population in a Real-World setting. Ann Intern Med.

[9] Centers for Disease Control and Prevention (CDC). COVID-19 after vaccination: possible breakthrough infection. 2022. https://www.cdc.gov/coronavirus/2019-ncov/vaccines/effectiveness/why-measure-effectiveness/breakthrough-cases.html. Accessed 1 Mar 2023.

[10] Jain VK, Iyengar KP, Ish P. Elucidating causes of COVID-19 infection and related deaths after vaccination. Diabetes & Metabolic Syndrome: Clinical Research & Reviews. 2021;15:102212. 10.1016/j.dsx.2021.102212 PMC8280649 34284226

[11] Külper-Schiek W, Piechotta V, Pilic A, Batke M, Dreveton L-S, Geurts B (2022). Facing the Omicron variant—how well do vaccines protect against mild and severe COVID-19? Third interim analysis of a living systematic review. Front Immunol.

[12] Yek C, Warner S, Wiltz JL, Sun J, Adjei S, Mancera A (2022). Risk factors for severe COVID-19 outcomes among persons aged ≥ 18 years who completed a primary COVID-19 Vaccination Series — 465 Health Care Facilities, United States, December 2020–October 2021. MMWR Morb Mortal Wkly Rep.

[13] Zhang L, Li Q, Liang Z, Li T, Liu S, Cui Q (2022). The significant immune escape of pseudotyped SARS-CoV-2 variant Omicron. Emerg Microbes Infections.

[14] YangYang, Gong X, Yang L, Li J, Zhang J, Wei L (2022). Regular and booster vaccination with inactivated vaccines enhance the neutralizing activity against Omicron variant both in the breakthrough infections and vaccinees. J Infect.

[15] Bundesministerium für Gesundheit. FAQs zum Impfen/ Auffrischungsimpfung. Zusammen gegen Corona. 2023. https://www.zusammengegencorona.de/faqs/impfen/auffrischungsimpfung/. Accessed 14 Mar 2023.

[16] Andrews N, Stowe J, Kirsebom F, Toffa S, Sachdeva R, Gower C (2022). Effectiveness of COVID-19 booster vaccines against COVID-19-related symptoms, hospitalization and death in England. Nat Med.

[17] Barda N, Dagan N, Cohen C, Hernán MA, Lipsitch M, Kohane IS (2021). Effectiveness of a third dose of the BNT162b2 mRNA COVID-19 vaccine for preventing severe outcomes in Israel: an observational study. Lancet.

[18] Doria-Rose NA, Shen X, Schmidt SD, O’Dell S, McDanal C, Feng W et al. Booster of mRNA-1273 strengthens SARS-CoV-2 Omicron neutralization. preprint. medRxiv; 2021.

[19] Hippisley-Cox J, Coupland CA, Mehta N, Keogh RH, Diaz-Ordaz K, Khunti K et al. Risk prediction of covid-19 related death and hospital admission in adults after covid-19 vaccination: national prospective cohort study. BMJ. 2021;:n2244. 10.1136/bmj.n2244 PMC8446717 34535466

[20] Amt für Stadtentwicklung und Statistik der Stadt Köln. Kölner Zahlenspiegel. Stadt Köln: Amt für Stadtenwicklung und Statistik; 2020. https://www.stadt-koeln.de/mediaasset/content/pdf15/statistik-standardinformationen/kölner_zahlenspiegel_2020_deutsch.pdf. Accessed 1 Mar 2023.

[21] Corona-in-Zahlen.de. Corona-Zahlen für Kreisfreie Stadt Köln. 2023. https://www.corona-in-zahlen.de/landkreise/sk köln/. Accessed 1 Mar 2023.

[22] Oberste M, Pusch L-M, Roth R, Shah-Hosseini K, Dewald F, Müller C (2021). Protocol of the Cologne Corona Surveillance (CoCoS) Study– a prospective population-based cohort study. BMC Public Health.

[23] Oberste M, Pusch L-M, Roth R, Shah-Hosseini K, Schmitz J, Heger E (2022). Results of the Cologne Corona surveillance (CoCoS) study– a prospective population-based cohort study: incidence data and potential underestimation of new SARS-CoV-2 adult infections by health authorities. BMC Public Health.

[24] Oberste M, Schnörch N, Shah-Hosseini K, Asenova T, Dewald F, Lehmann C (2023). Results of the Cologne Corona Surveillance (CoCoS) study– a cross-sectional study: survey data on risk factors of SARS-CoV-2 infection in adults. BMC Public Health.

[25] National Institutes of Health. Clinical spectrum of SARS-CoV-2 infection. Coronavirus disease 2019 (COVID-19) treatment guidelines. 2022. https://www.covid19treatmentguidelines.nih.gov/overview/clinical-spectrum/. Accessed 1 Mar 2023. 34003615

[26] Robert Koch-Institut. Wöchentlicher Lagebericht des RKI zur Coronavirus-Krankheit-2019 (COVID-19). Berlin: Robert-Koch-Institut.; 2022. https://www.rki.de/DE/Content/InfAZ/N/Neuartiges_Coronavirus/Situationsberichte/Wochenbericht/Wochenbericht_2022-01-20.pdf?__blob=publicationFile. Accessed 1 Mar 2023.

[27] Bayerisches Landesamt für Gesundheit und Lebensmittelsicherheit, Robert Koch-Institut (2020). Beschreibung Des Bisherigen Ausbruchsgeschehens Mit dem neuartigen Coronavirus SARS-CoV-2 in Deutschland (stand: 12. Februar 2020). Epid Bull.

[28] Zhu Y, Liu S, Zhang D (2022). Effectiveness of COVID-19 vaccine Booster Shot compared with Non-booster: a Meta-analysis. Vaccines.

[29] Belik M, Jalkanen P, Lundberg R, Reinholm A, Laine L, Väisänen E (2022). Comparative analysis of COVID-19 vaccine responses and third booster dose-induced neutralizing antibodies against Delta and Omicron variants. Nat Commun.

[30] Liu X, Huang J, Li C, Zhao Y, Wang D, Huang Z (2021). The role of seasonality in the spread of COVID-19 pandemic. Environ Res.

[31] Adedokun KA, Olarinmoye AO, Mustapha JO, Kamorudeen RT (2020). A close look at the biology of SARS-CoV-2, and the potential influence of weather conditions and seasons on COVID-19 case spread. Infect Dis Poverty.

[32] Lund LC, Støvring H, Pottegård A, Andersen M, Hallas J (2023). Cox regression using a calendar time scale was unbiased in simulations of COVID-19 vaccine effectiveness & safety. J Clin Epidemiol.

[33] Paul-Ehrlich-Institut. COVID-19 Vaccines. Paul-Ehrlich-Institut; 2023. https://www.pei.de/EN/medicinal-products/vaccines-human/covid-19/covid-19-list-1.html Accessed 13 Nov 2023.

[34] Zhang S, Yang Z, Li Z-N, Chen Z-L, Yue S-J, Fu R-J (2022). Are older people really more susceptible to SARS-CoV-2?. Aging Disease.

[35] Sharma A, Oda G, Holodniy M. COVID-19 Vaccine breakthrough infections in veterans health administration. preprint. medRxiv. 2021; 10.1101/2021.09.23.21263864

[36] Uschner D, Bott M, Lagarde WH, Keating J, Tapp H, Berry AA (2022). Breakthrough SARS-CoV-2 infections after vaccination in North Carolina. Vaccines.

[37] Stouten V, Hubin P, Haarhuis F, van Loenhout J, Billuart M, Brondeel R (2022). Incidence and risk factors of COVID-19 vaccine breakthrough infections: a prospective cohort study in Belgium. Viruses.

[38] Proesmans K, Hancart S, Braeye T, Klamer S, Robesyn E, Djiena A et al. COVID-19 contact tracing in Belgium: main indicators and performance, January - September 2021. preprint. Research Square. 2022; 10.21203/rs.3.rs-1326456/v1 10.1186/s13690-022-00875-6 PMC9005619 35418097

[39] Daoust J-F (2020). Elderly people and responses to COVID-19 in 27 countries. PLoS ONE.

[40] Ben Fredj S, Ghammem R, Zammit N, Maatouk A, Haddad N, Haddad N (2022). Risk factors for severe Covid-19 breakthrough infections: an observational longitudinal study. BMC Infect Dis.

[41] Berry CE, Han MK, Thompson B, Limper AH, Martinez FJ, Schwarz MI (2015). Older adults with chronic lung Disease Report Less Limitation compared with younger adults with similar lung function impairment. Annals ATS.

[42] Tack M, Altose MD, Cherniack NS (1982). Effect of aging on the perception of resistive ventilatory loads. Am Rev Respir Dis.

[43] Allen SC, Khattab A (2006). The tendency to altered perception of airflow resistance in aged subjects might be due mainly to a reduction in diaphragmatic proprioception. Med Hypotheses.

[44] Peckham H, de Gruijter NM, Raine C, Radziszewska A, Ciurtin C, Wedderburn LR (2020). Male sex identified by global COVID-19 meta-analysis as a risk factor for death and ITU admission. Nat Commun.

[45] Pivonello R, Auriemma RS, Pivonello C, Isidori AM, Corona G, Colao A (2021). Sex disparities in COVID-19 severity and outcome: are men weaker or women stronger?. Neuroendocrinology.

[46] Lamprecht B, Vanfleteren LE, Studnicka M, Allison M, McBurnie MA, Vollmer WM (2013). Sex-related differences in respiratory symptoms: results from the BOLD study. Eur Respir J.

[47] Chen W, Woods SL, Puntillo KA (2005). Gender differences in symptoms associated with acute myocardial infarction: a review of the research. Heart Lung.

[48] Wilder-Smith A, Frahsa A (2022). Impact of BMI on COVID-19 vaccine effectiveness. Lancet Diabetes Endocrinol.

[49] Gao M, Piernas C, Astbury NM, Hippisley-Cox J, O’Rahilly S, Aveyard P (2021). Associations between body-mass index and COVID-19 severity in 6·9 million people in England: a prospective, community-based, cohort study. Lancet Diabetes Endocrinol.

[50] de Leeuw AJM, Oude Luttikhuis MAM, Wellen AC, Müller C, Calkhoven CF (2021). Obesity and its impact on COVID-19. J Mol Med.

[51] Frankenfield DC, Rowe WA, Cooney RN, Smith JS, Becker D (2001). Limits of body mass index to detect obesity and predict body composition. Nutrition.

[52] Jitnarin N, Poston WSC, Haddock CK, Jahnke S, Tuley BC (2013). Accuracy of body mass index-defined overweight in fire fighters. Occup Med.

[53] Alqahtani JS, Oyelade T, Aldhahir AM, Alghamdi SM, Almehmadi M, Alqahtani AS (2020). Prevalence, severity and mortality associated with COPD and smoking in patients with COVID-19: a Rapid systematic review and Meta-analysis. PLoS ONE.

[54] Groskreutz DJ, Monick MM, Babor EC, Nyunoya T, Varga SM, Look DC (2009). Cigarette smoke alters respiratory Syncytial Virus–Induced apoptosis and replication. Am J Respir Cell Mol Biol.

[55] McAlinden KD, Eapen MS, Lu W, Chia C, Haug G, Sohal SS (2020). COVID-19 and vaping: risk for increased susceptibility to SARS-CoV-2 infection?. Eur Respir J.

[56] Beltramo G, Cottenet J, Mariet A-S, Georges M, Piroth L, Tubert-Bitter P (2021). Chronic respiratory diseases are predictors of severe outcome in COVID-19 hospitalised patients: a nationwide study. Eur Respir J.

[57] Aveyard P, Gao M, Lindson N, Hartmann-Boyce J, Watkinson P, Young D (2021). Association between pre-existing respiratory disease and its treatment, and severe COVID-19: a population cohort study. Lancet Respiratory Med.

[58] Polverino F, Kheradmand F (2021). COVID-19, COPD, and AECOPD: immunological, epidemiological, and clinical aspects. Front Med.

[59] Robert Koch-Institut. Impfung bei Kindern und Jugendlichen (Stand: 7.2.2023). Robert Koch Institut. 2023. https://www.rki.de/SharedDocs/FAQ/COVID-Impfen/FAQ_Liste_Impfung_Kinder_Jugendliche.html. Accessed 2 Mar 2023.

