# Supplementary material for: Results of the Cologne Corona Surveillance (CoCoS) project– a cross-sectional study: survey data on risk factors of SARS-CoV-2 infection, and moderate-to-severe course in primarily immunized adults
Source: BMC Public Health. 2024 Feb 21;24:548. doi: 10.1186/s12889-024-17958-4 (PMC10882740; doi:10.1186/s12889-024-17958-4)

**Supplementary Table 1** Multicollinearity assessment concerning multivariable analysis of associations with breakthrough infection using tolerance/Variance Inflation Factor (VIF)

| **Coefficients** | | | |  |
| --- | --- | --- | --- | --- |
| Model | |  |  |  |
|  |  | Tolerance | Variance Inflation Factor (VIF) | |
|  |  |  |  | |
|  | Age | .745 | 1.343 | |
|  | Sex | .973 | 1.028 | |
|  | Body-Mass-Index | .879 | 1.138 | |
|  | Vaccination status | .964 | 1.037 | |
|  | Smoking | .986 | 1.014 | |
|  | Chronic lung disease | .979 | 1.021 | |
|  | Cardiovascular disease | .721 | 1.387 | |
|  | Immunodeficiency | .966 | 1.035 | |
|  | Cancer present or treated in the last year | .956 | 1.046 | |

**Supplementary Table 2** Multicollinearity assessment concerning multivariable analysis of associations with severity of breakthrough infection using tolerance/Variance Inflation Factor (VIF)

| **Coefficients** | | | |  |
| --- | --- | --- | --- | --- |
| Model | |  |  |  |
|  |  | Tolerance | Variance Inflation Factor (VIF) | |
|  |  |  |  | |
|  | Age | .758 | 1.319 | |
|  | Sex | .973 | 1.028 | |
|  | Body-Mass-Index | .880 | 1.137 | |
|  | Vaccination status | .966 | 1.035 | |
|  | Smoking | .987 | 1.013 | |
|  | Chronic lung disease | .986 | 1.014 | |
|  | Cardiovascular disease | .722 | 1.385 | |

**Supplementary Figure 1** Associations with vaccination breakthroughs – Forest plots of the results of the univariable and multivariable Cox proportional hazards survival regression


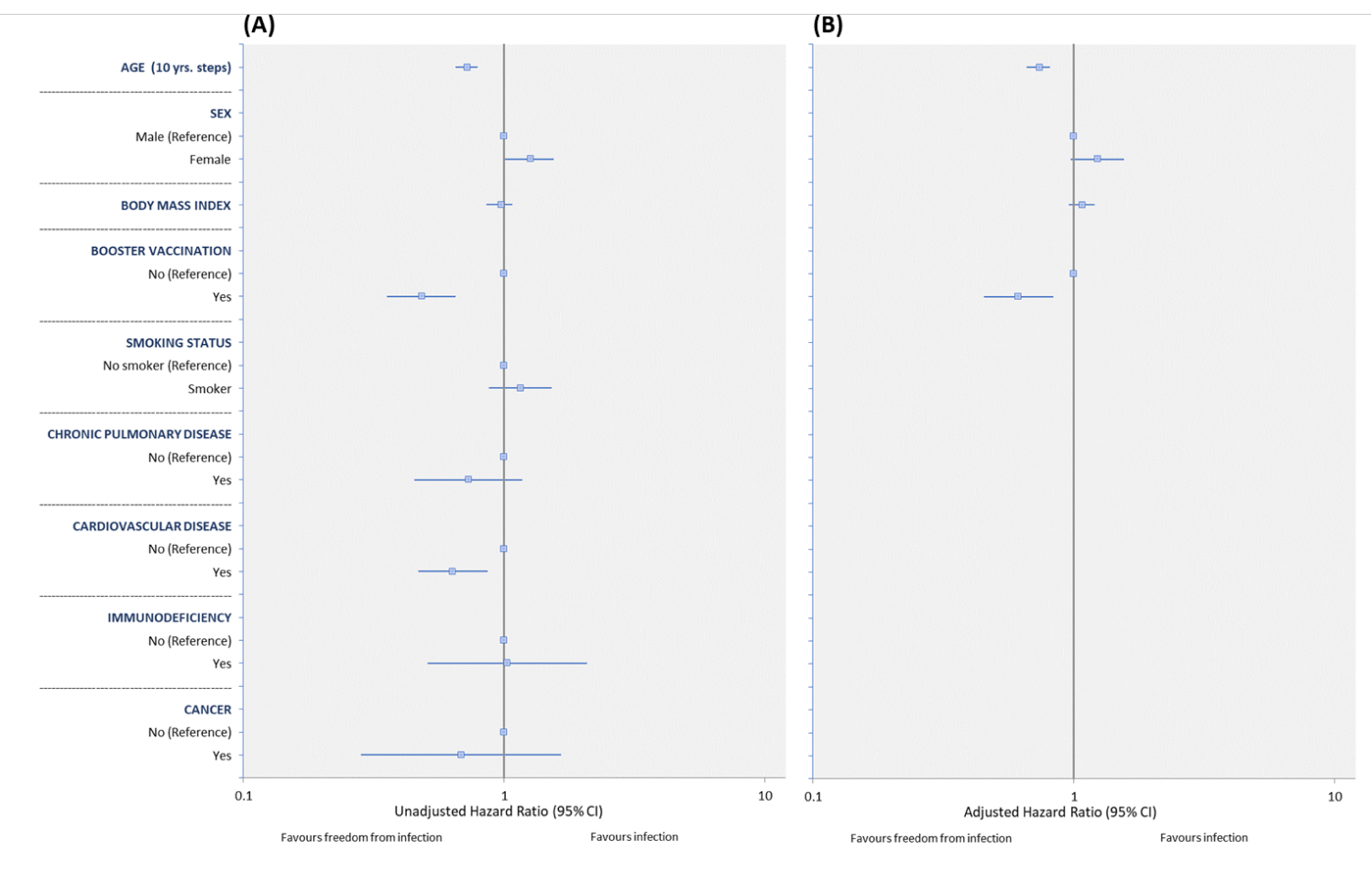


**Supplementary Figure 2** Associations with moderate-to-severe course of SARS-CoV-2 – Forest plots of the results of the univariable and multivariable Fine and Gray subdistribution hazard regression


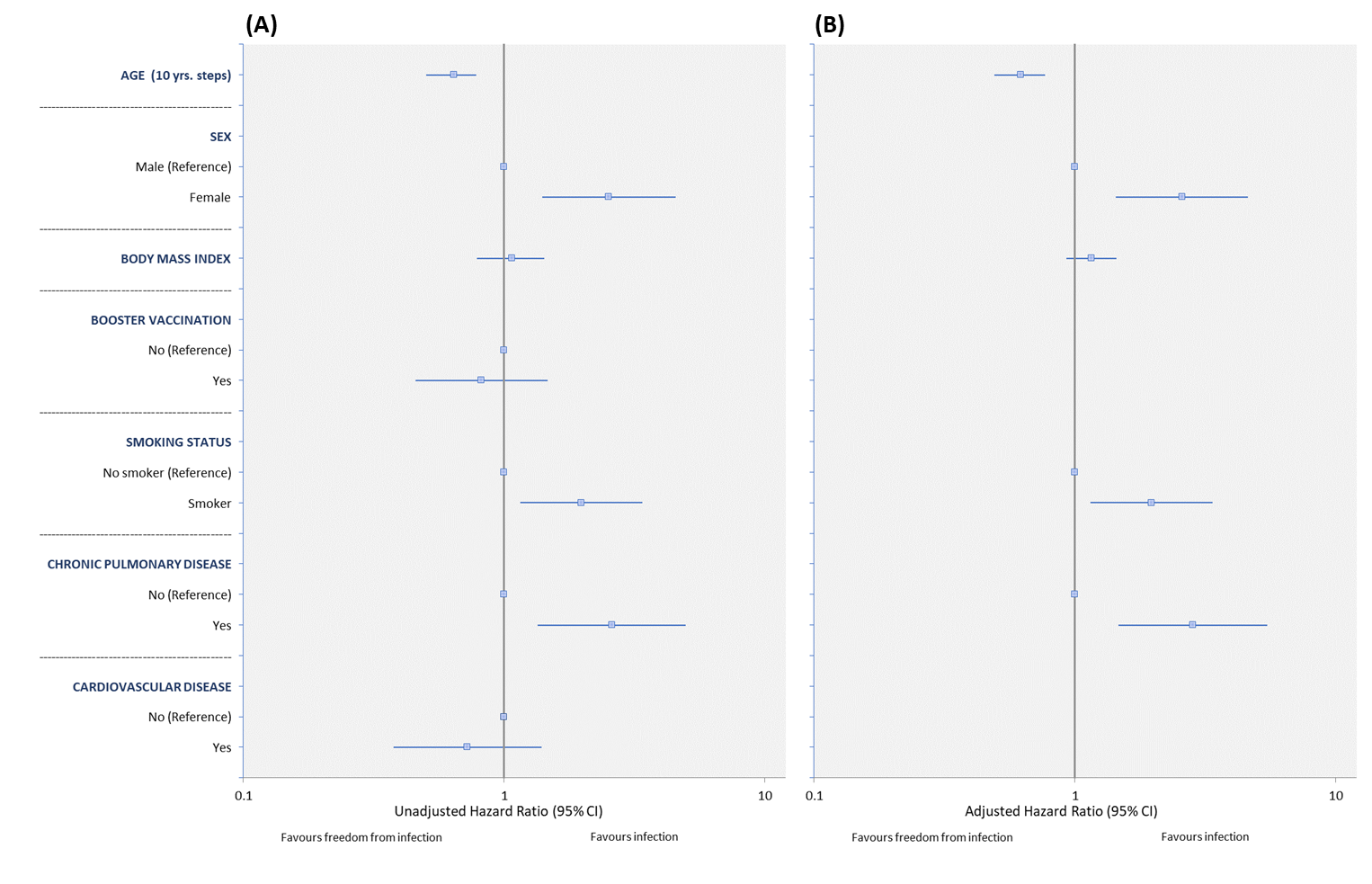

Supplement: Supplementary file 1 — Supplementary Material 1 [file 12889_2024_17958_MOESM1_ESM.docx]
